# Identification of Cardiomyopathy-Associated Circulating miRNA Biomarkers in Muscular Dystrophy Female Carriers Using a Complementary Cardiac Imaging and Plasma Profiling Approach

**DOI:** 10.3389/fphys.2018.01770

**Published:** 2018-12-21

**Authors:** Anca Florian, Alexandru Patrascu, Roman Tremmel, Sabine Rösch, Udo Sechtem, Matthias Schwab, Elke Schaeffeler, Ali Yilmaz

**Affiliations:** ^1^Department of Cardiology, University Hospital Münster, Münster, Germany; ^2^Division of Cardiology, Robert-Bosch-Hospital, Stuttgart, Germany; ^3^Dr. Margarete Fischer-Bosch Institute of Clinical Pharmacology, Stuttgart, Germany; ^4^Department of Clinical Pharmacology, Institute of Experimental and Clinical Pharmacology and Toxicology, University Hospital Tübingen, Tübingen, Germany; ^5^Department of Pharmacy and Biochemistry, University of Tübingen, Tübingen, Germany

**Keywords:** cardiomyopathy, muscular dystrophy, female carriers, microRNA, cardiovascular magnetic resonance

## Abstract

**Background:** Different from males with Duchenne/Becker muscular dystrophy (DMD/BMD) in whom overt myopathy is the rule, muscular dystrophy (MD) female carriers are mostly free of skeletal muscle symptoms. However, similar to MD males, these females are also prone to cardiomyopathy. Since circulating microRNAs (miRNAs) have been proposed as diagnostic biomarkers for various cardiovascular diseases, the aim of the current study was to identify specific circulating miRNAs in the plasma of female DMD/BMD carriers that may allow an early and accurate diagnosis of cardiac involvement in these cases.

**Methods:** Twenty-nine female MD carriers and 24 age-matched healthy female controls were prospectively enrolled. All MD carriers and controls underwent comprehensive cardiovascular magnetic resonance (CMR) studies as well as venous blood sampling on the same day.

**Results:** An impaired left ventricular (LV) systolic function was detected in 4 (14%) MD carriers while late gadolinium enhancement (LGE) indicative of myocardial fibrosis was present in 13 female patients (45%)—with an exclusively non-ischemic pattern. Among the circulating miRNAs examined, six were significantly up-regulated in MD carriers compared to female controls: miR-206 (103-fold increase, *p* < 0.0001), miR-222 (41-fold, *p* < 0.0001), miR-26a (fourfold, *p* = 0.029), miR-342 (27-fold, *p* < 0.0001), miR-378a-3p (minimum 3,600-fold; almost undetectable in controls, *p* = 0.013), miR-378a-5p (64-fold, *p* < 0.0001); only two miRNAs were substantially down-regulated in MD carriers: miR-144 (*p* < 0.0001) and miR-29a (*p* = 0.002) (both undetectable in carriers). A significant down-regulation of the miR-29c (<0.001-fold, *p* = 0.006) was observed in MD carriers with abnormal CMR findings (comprising functional and/or structural abnormalities) compared to those with normal CMR examinations. Univariable analyses regarding the presence of abnormal CMR findings resulted in four significant variables: LV end-diastolic volume index (EDVi), LV end-systolic volume index (ESVi), an elevated plasma creatine kinase (CK), and decreased serum miR-29c levels. In subsequent multivariable analysis, the only independent predictor for an abnormal CMR among MD carriers was circulating miR-29c (OR 0.99, 95% CI 0.98–0.99, *p* = 0.037). Moreover, an elevated CK and/or a downregulated miR-29c level (<0.05 × 10^-3^) resulted in an improved AUC value of 0.79 (0.62–0.97, *p* = 0.007) (79, 80 and 80%, sensitivity, specificity and overall accuracy) for the CMR-based diagnosis of cardiomyopathy in MD carriers when compared to using the two parameters individually.

**Conclusion:** In female MD carriers, down-regulation of circulating miR-29c relates to the presence of functional and/or structural cardiac abnormalities (as detected by CMR) and appears to be a promising novel biomarker—in addition to conventional CK plasma levels—for an early diagnosis of cardiomyopathy.

## Introduction

Duchenne and Becker muscular dystrophies are X-linked genetic disorders affecting dystrophin synthesis or function (a major sarcolemmal protein) and leading to progressive muscular disease with proximal skeletal as well as cardiac involvement (Verhaert et al., 2011; Yilmaz and Sechtem, 2012). In MD males, a MD-related cardiomyopathy evolves with a characteristic, non-ischemic pattern of LV myocardial fibrosis leading to dilated cardiomyopathy, heart failure and ventricular arrhythmias (Finsterer and Stollberger, 2005; Hermans et al., 2010; Mavrogeni et al., 2010; Yilmaz and Sechtem, 2012).

While in female MD-carriers relevant skeletal muscle symptoms are rarely observed, cardiomyopathy with the same cardiac phenotype as in their male counterparts frequently occurs—particularly present in DMD female carriers (Hoogerwaard et al., 1999; Finsterer and Stollberger, 2005; Schade van Westrum et al., 2011; Mavrogeni et al., 2013; Florian et al., 2016). Noteworthy, cardiac involvement may lead to overt heart failure symptoms in approximately 10% of female MD-carriers, sometimes even necessitating cardiac transplantation (Melacini et al., 1998; Barison et al., 2009; Schade van Westrum et al., 2013; Wexberg et al., 2016).

Today, CMR is a well-established and reliable tool for the early and sensitive diagnosis of cardiac involvement not only in male MD patients but also in female MD-carriers, mainly due to the possibility for a non-invasive myocardial tissue characterization with detection of even subtle forms of myocardial fibrosis by LGE-imaging or T1-mapping techniques (Silva et al., 2007; Yilmaz et al., 2008, 2010; Mavrogeni et al., 2013; Florian et al., 2014b,c; Giglio et al., 2014; Wexberg et al., 2016).

MicroRNAs (miRNAs) are small, both intracellular and circulating non-coding molecules and can modify gene expression and thereby regulate essential cellular functions (Quiat and Olson, 2013). In the context of recent advances in molecular diagnostics, there is a growing body of evidence that circulating miRNAs can be used as diagnostic as well as prognostic biomarkers for a wide range of disorders—including muscular and cardiovascular diseases (Gupta et al., 2010; Quiat and Olson, 2013; Romaine et al., 2015; Becker et al., 2016; Israeli et al., 2016).

The aim of the current study was to identify specific circulating miRNAs in the plasma of female DMD/BMD carriers that may allow an early and accurate diagnosis of cardiac involvement in these cases.

## Materials and Methods

### Study Population

Twenty-nine females with genetically confirmed DMD or BMD carrier status (“MD-carriers,” 18 DMD and 11 BMD, Supplementary Table 1) and 24 age-matched healthy females (“Female controls”) were prospectively enrolled between 2009 and 2015 and underwent comprehensive CMR studies as well as venous blood sampling, as described elsewhere (Becker et al., 2016; Florian et al., 2016). In addition, 25 age- and disease subtype-matched males with known MD (“MD patients,” 12 DMD and 13 BMD) as well as 26 age-matched healthy males (“Male controls”), part of an already published study and in whom the same protocol was carried out, served as additional groups for comparative analyses (Becker et al., 2016).

All patients underwent a thorough neurological and cardiac examination. The female MD-carriers were considered as having a manifest form of neuromuscular disease whenever skeletal muscle symptoms, i.e., weakness and/or muscle pain, were present (Soltanzadeh et al., 2010). The presence of cardiac symptoms included at least one of the following: chest pain, dyspnoea and/or palpitations. The study protocol complies with the Declaration of Helsinki and was approved by the local ethics committee (Landesärztekammer Stuttgart). Informed consent was obtained from the patients prior to study inclusion.

### Blood Sampling in Patients and Controls

EDTA blood samples were collected on the same day of the CMR study and plasma aliquots were harvested and stored at -20°C as previously described (Becker et al., 2016). Both in the study groups and controls, laboratory determinations for cardiac biomarkers—high sensitive troponin I (hs-Trop) and NT-proBNP—were performed using standard methods. In addition, CK levels were determined as biomarker of skeletal muscle damage. The cardiac and skeletal muscle biomarkers were considered elevated when serum levels exceeded the upper laboratory reference limit.

### miRNA Extraction and Quantification

RNA was extracted from 400 μl of plasma using mirVana miRNA isolation kit (Life technologies, Carlsbad, CA, United States) following the manufacturer’s protocol, eluted in 75 μl elution solution and stored at -80°C. miRNA selection for quantification was based on literature data related to either cardiovascular diseases and/or DMD/BMD were reverse transcribed using TaqMan MicroRNA Reverse Transcription Kit (Life technologies) (Becker et al., 2016). Individual stem-loop reverse transcription primers included in the predeveloped TaqMan miRNA assay (Life technologies) were pooled at a final dilution of 0.05× for each primer. The final reaction volume of 7.5 μl contained 0.15 μl 100 mM dNTP, 1.5 μl multiscribe reverse transcriptase (50 U/μl), 0.75 μl 10× RT buffer, 0.095 μl RNase inhibitor (20 U/μl), 3 μl primer pool and 2 μl of total RNA. The reaction was performed following conditions of manufacturer. To improve sensitivity of miRNA quantification, a pre-amplification reaction was performed. TaqMan miRNA assays included in the TaqMan miRNA assay (Life technologies) were pooled at a final dilution of 0.2× for each assay. Pre-amplification reaction was done at 10 μl final volume containing 5 μl TaqMan PreAmp Master Mix (2×), 1.5 μl of assay pool, 2.5 μl of nuclease-free water and 1 μl of cDNA. The pre-amplification PCR was run according to the manufacturer’s protocol, the pre-amplification PCR product was diluted 1:5 with suspension buffer (Teknova AS, Hollister, CA, United States) and stored at -20°C until need. The miRNA expression levels were quantified by realtime PCR using TaqMan^®^ Universal Master Mix II (no UNG) and TaqMan miRNA assays (Life technologies) on a real-time PCR BioMark system (Fluidigm Corporation, South San Francisco, CA, United States) following the manufacturer’s protocol. Relative levels of miRNA expression were calculated by normalization to expression levels of miR-16 and thereafter multiplied by 103 in order to increase readability in the respective tables. The following miRNAs had to be excluded from final analysis due to failing measurements: miR-1, miR-31, miR-34c, miR-95-3p, miR-133a, miR-208a-3p, miR-208b-3p, miR-499a-3p, miR-499a-5p and miR-539-5p.

### CMR Data Acquisition

Electrocardiographic-gated CMR studies were performed on a 1.5-T scanner (Aera, Siemens Medical Solutions, Erlangen, Germany) using commercially available cardiac software, electrocardiographic triggering, and cardiac-dedicated surface coils. Cine-imaging was performed using a steady-state-free-precession sequence in three long-axis slices (four-, three- and two-chamber) and a stack of short-axis slices completely covering the LV. LGE-imaging was performed using a T1-weighted inversion recovery gradient-echo sequence 10–15 min after intravenous contrast administration (0.15 mmol/kg Magnevist^®^) in the same imaging planes as the cine-images.

### CMR Data Analysis

Cardiovascular magnetic resonance analysis was performed off-line by two experienced readers blinded to gender and clinical characteristics as described elsewhere (Florian et al., 2014a). An abnormal CMR was defined by at least one of the following: (1) functional abnormalities: LV ejection fraction (LV-EF) <55% and/or an RV ejection fraction (RV-EF) <45%; (2) structural abnormalities: LGE presence in at least one myocardial segment.

### Statistical Analysis

Continuous variables are expressed as mean ± SD. Skewed variables are expressed as median and interquartile range (IQR). Categorical variables are expressed as frequency with percentage. Student’s *t*-tests were used for comparison of normally distributed variables, while Mann–Whitney *U* tests were used for comparison of non-normally distributed variables. Non-parametric Kruskal–Wallis tests with Bonferroni *post-hoc* correction were used in case of multiple comparisons of non-normally distributed variables. Chi-square tests with Yate’s correction were used to compare categorical variables expressed as proportions. Parametric Pearson or non-parametric Spearman correlations were used as corresponded for correlation analysis. In order to find independent predictors for abnormal CMR findings, i.e., LGE presence, we performed a univariable regression analysis first. Subsequently, the parameters with significant *p*-values were introduced into the multivariable regression analysis. Extremely skewed miRNAs (skewness statistic <-2 or >2) were Log_10_ transformed before introduced in the regression analysis. Finally, receiver operating characteristic curves (ROC) were analyzed to assess specificity and sensitivity for plasma biomarkers. Statistical analysis was performed using SPSS software for Windows (version 24, SPSS, Chicago, IL, United States). A *p*-value ≤0.05 was considered statistically significant.

## Results

### Characteristics of the Study Population

The general characteristics for the MD-carrier (*N* = 29) and female control (*N* = 24) groups are shown in Table 1. Eighteen of the MD-carriers (62%) had a DMD and 11 (38%) had a BMD carrier status.

**Table 1:** Study population characteristics and CMR parameters.

	MD-carrier	Female controls	*p*-value^∗∗^
	*N* = 29	*N* = 24	
Female, *n* (%)	29 (100)	24 (100)	1.00
Age, years [mean ± SD]	45 ± 14	41 ± 11	0.38
DMD/BMD, *n* (%)	18 (62)/11 (38)	–	
BMI, [mean ± SD] kg/m^2^	25 ± 4	24 ± 3	0.09
Skeletal muscle symptoms, *n* (%)	4 (14)	0 (0)	0.12
Cardiac symptoms, *n* (%)	9 (31)	0 (0)	**0.003**
ACE inhibitor/ARB, *n* (%)	2 (7)	0 (0)	0.49
Beta blocker, *n* (%)	3 (10)	0 (0)	0.24
**CMR findings**			
LV-EDVi, [mean ± SD] (ml/m^2^)	71 ± 18	65 ± 11	0.15
LV-ESVi, [mean ± SD] (ml/m^2^)	26 ± 9	23 ± 5	0.20
LV-massi, [mean ± SD] (g/m^2^)	48 ± 9	43 ± 6	**0.022**
LV-EF, [mean ± SD] (%)	64 ± 7	65 ± 4	0.47
RV-EF, [mean ± SD] (%)	55 ± 11	56 ± 8	0.87
LV-EF < 55%, *n* (%)	4 (14)	0 (0)	0.12
RV-EF < 45%, *n* (%)	4 (14)	0 (0)	0.12
LGE presence, *n* (%)	13 (45)	0 (0)	**<0.0001**
LGE extent, *n* (%)	0 (0–7)	–	
Abnormal CMR, *n* (%)	14 (48)	0 (0)	**<0.0001**
**Serum measurements**			
CK, U/L [median (IQR)]	168 (111–595)	103 (80–149)	**0.004**
Elevated CK, *n* (%)^∗^	14 (48)	1 (4)	**<0.0001**
Elevated hs-Trop, *n* (%)^†^	2 (7)	0 (0)	0.49
Elevated NT-proBNP, *n* (%)^‡^	2 (7)	0 (0)	0.49

*^∗^ >190 U/l. ^†^ >14 pg/ml. ^‡^ >450 pg/ml. ^∗∗^Student’s *t*-test, Mann–Whitney *U* test, Kruskal–Wallis test with Bonferroni *post hoc* correction or Chi-square test were used appropriate. Bold values indicate significant *p* < 0.05*.

MD-carriers suffered more frequently from cardiac symptoms (31 vs. 0%, *p* = 0.003)—but not from muscular skeletal symptoms (14 vs. 0%, *p* = NS) —in comparison to female controls. Moreover, MD-carriers had significantly higher CK levels but no significant cardiac biomarker elevation when compared to controls.

### CMR Findings in MD-Carriers and Female Controls

The detailed CMR results for female MD-carriers and female controls are given in Table 1. Three female carriers demonstrated an isolated impairment in LV function (LV-EF < 55%), another three an impairment in RV function (RV-EF < 45%) and one carrier an impairment in both LV and RV function, however, all being mild in degree. LV mass index was slightly but significantly higher in female MD carriers vs. female controls, but none of the members of both groups showed substantial LV hypertrophy, according to current criteria (Kawel-Boehm et al., 2015).

Presence of LGE indicative of myocardial fibrosis was detected in 13 (45%) of the female MD-carriers (*p* < 0.0001 vs. female controls)—with an exclusively non-ischemic pattern. In LGE-positive carriers, median LGE extent was 7% (IQR 5–16%) of LV mass.

When considering functional and/or structural abnormalities, a pathological CMR finding was observed in 14 (48%) of the female MD-carriers whereas all females in the control group demonstrated normal CMR examinations (*p* < 0.0001).

**FIGURE 1:**
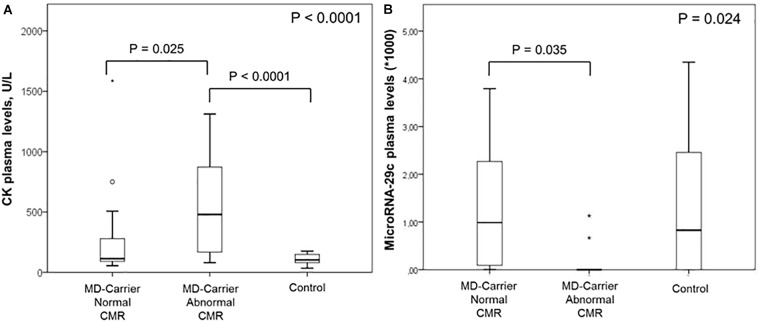
Box plots for plasma levels of plasma CK **(A)** and miR-29c **(B)** in female carriers with and without cardiovascular magnetic resonance (CMR) abnormalities as well as in healthy controls.

Patient and CMR characteristics according to presence of isolated functional or structural CMR abnormalities are further detailed in Table 2. LGE prevalence and extent were significantly higher in female MD-carriers with an impaired LV and/or RV systolic function. Interestingly, female MD-carriers with functional and/or structural CMR abnormalities had significantly higher CK levels when compared to female controls and to MD-carriers with a normal CMR, respectively (Table 2 and Figure 1A). The prevalence of an elevated total CK (*p* = 0.009) as well as of a DMD carrier status (*p* = 0.05)—but not of elevated cardiac biomarkers—were significantly higher in female MD carriers with LGE presence compared to those without. Moreover, a moderate positive correlation between LGE extent and CK plasma levels was detected (Spearman-Rho +0.514, *p* = 0.004).

**Table 2:** Patient characteristics and cardiac findings according to CMR, functional and structural results.

	Normal CMR	Abnormal CMR	*p*-value	Normal LV & RV function	Impaired LV &/or RV function	*p*-value	LGE-negative *N* = 16	LGE-positive *N* = 13	*p*-value
	*N* = 15	*N* = 14		*N* = 22	*N* = 7				
Female, *n* (%)	15 (100)	14 (100)	1.00	22 (100)	7 (100)	1.00	16 (100)	13 (100)	1.00
Age, years	49 ± 13	40 ± 13	0.10	45 ± 14	44 ± 12	0.89	48 ± 13	40 ± 14	0.11
DMD/BMD, *n* (%)	7 (47)/8 (53)	11 (79)/3(21)	0.13	12 (55)/10 (46)	6 (86)/1 (14)	0.20	7 (44)/9 (56)	11 (85)/2 (15)	**0.05**
BMI, kg/m^2^	25 ± 4	26 ± 4	0.74	25 ± 4	26 ± 5	0.66	25 ± 4	25 ± 4	0.99
Skeletal muscle symptoms, *n* (%)	1 (7)	3 (21)	0.33	2 (9)	2 (29)	0.24	1 (6)	3 (23)	0.30
Cardiac symptoms, *n* (%)	3 (20)	6 (43)	0.18	5 (23)	4 (57)	0.16	3 (19)	6 (46)	0.23
LV-EDVi, ml/m^2^	64 ± 17	79 ± 15	**0.027**	69 ± 18	80 ± 15	0.15	65 ± 17	79 ± 16	**0.03**
LV-ESVi, ml/m^2^	22 ± 6	30 ± 10	**0.026**	23 ± 7	34 ± 9	**0.003**	23 ± 7	30 ± 10	0.06
LV-mass, g/m^2^	48 ± 9	48 ± 10	0.91	47 ± 8	50 ± 13	**0.003**	48 ± 9	48 ± 10	0.97
LV-EF, %	65 ± 5	62 ± 8	0.29	66 ± 6	57 ± 7	**0.002**	64 ± 6	63 ± 8	0.59
RV-EF, %	58 ± 11	52 ± 10	0.17	58 ± 10	45 ± 8	**0.003**	57 ± 11	52 ± 11	0.22
LV-EF < 55%, *n* (%)	0 (0)	4 (29)	**0.042**	0 (0)	4 (57)	**0.001**	1 (6)	3 (23)	0.29
RV-EF < 45%, *n* (%)	0 (0)	4 (29)	**0.042**	0 (0)	4 (57)	**0.001**	0 (0)	4 (31)	**0.030**
LGE presence, *n* (%)	0 (0)	13 (93)	**<0.0001**	7 (32)	6 (86)	**0.026**	–	7 (5–16)	
LGE extent, %	–	7 (4–16)		0 (0–4)	7 (4–13)	**0.024**	1 (6)	13 (100)	**<0.001**
Elevated CK, *n* (%)^∗^	4 (27)	10 (71)	**0.027**	10 (46)	4 (57)	0.68	4 (25)	10 (77)	**0.009**
Elevated hs-Trop, *n* (%)^†^	1 (7)	1 (7)	1.00	1 (5)	1 (14)	0.43	1 (6)	1 (8)	1.00
Elevated NT-proBNP, *n* (%)^‡^	0 (0)	2 (14)	0.22	1 (5)	1 (14)	0.43	0 (0)	2 (15)	0.19

*^∗^ >190 U/l. ^†^ >14 pg/ml. ^‡^ >450 pg/ml. Bold values indicate significant *p* < 0.05*.

### Gender and DMD/BMD Related Differences in miRNA Expression

In order to assess potential gender- and disease subtype-related differences in miRNA expression, we first compared circulating miRNA levels in the female control group with an age-matched male control group (*N* = 26). As shown in the Supplementary Table 2, no significant differences in plasma levels for the 17 selected miRNAs were found between healthy females and males. Secondly, we compared the miRNA levels in female MD-carriers to an age- and DMD/BMD-matched male group (*N* = 25). As shown in the Supplementary Table 3, miR-29c levels were significantly higher (*p* = 0.024) and miR-144 was undetectable, (*p* = 0.001) in female MD-carriers compared to MD males.

### miRNA Findings in Female MD-Carriers and Female Controls

Serum miRNA results in female MD carriers and female controls are shown in Table 3. A significant up-regulation in female MD carriers compared to female controls was found for 6 of the 17 plasma miRNAs that were successfully examined: miR-206 (103-fold increase, *p* < 0.0001), miR-222 (41-fold, *p* < 0.0001), miR-26a (fourfold, *p* = 0.029), miR-342 (27-fold, *p* < 0.0001), miR-378a-3p (minimum 3,600-fold; almost undetectable in controls, *p* = 0.013), miR-378a-5p (64-fold, *p* < 0.0001). Additionally, a significant down-regulation was found for two miRNAs: miR-144 (*p* < 0.0001) and miR-29a (*p* = 0.002), both undetectable in the plasma of MD-carriers.

**Table 3:** Serum miRNA results in MD-carriers vs. controls.

miRNA	MD-carrier	Female controls	*p*-value
serum levels^∗^ (×10^3^)	*N* = 29	*N* = 24	
-206	38.73 (10.86–70.06)	0.38 (0.13–0.87)	**<0.0001**
-144	0.00 (0.00–0.00)	12.65 (9.12–21.03)	**<0.0001**
-146b-5p	0.00 (0.00–95.45)	19.88 (6.41–42.27)	0.35
-15b	0.00 (0.00–13.13)	2.75 (1.24–8.49)	0.21
-195	9.99 (0.58–20.91)	13.46 (11.74–15.91)	0.20
-20b	35.91 (11.55–66.22)	41.51 (31.65–66.94)	0.22
-21-5p	2.92 (0.00–77.28)	10.55 (7.06–25.90)	0.40
-221	0.00 (0.00–32.99)	13.59 (0.89–35.06)	0.06
-222	3972.80 (1489.47–6881.28)	95.65 (67.93–149.18)	**<0.0001**
-26a	191.59 (40.72–474.63)	52.50 (17.12–136.09)	**0.029**
-29a	0.00 (0.00–0.00)	0.93 (0.00–2.01)	**0.002**
-29c	0.09 (0.00–1.64)	0.83 (0.00–2.32)	0.42
-342	2633.27 (1194.51–4321.52)	98.71 (66.63–142.78)	**<0.0001**
-378a-3p	3.59 (0.00–88.53)	0.00 (0.00–0.34)	**0.013**
-378a-5p	53.57 (18.76–170.52)	0.85 (0.48–1.55)	**<0.0001**
-451	144.0 (0.00–244.83)	124.83 (103.12–185.29)	0.68
-93	13.50 (0.00–55.57)	38.71 (27.66–46.52)	0.11

*^∗^Each miRNA serum level was normalized to miRNA-16 serum levels and thereafter multiplied by 10^*3*^ in order to increase readability. Bold values indicate significant *p* < 0.05*.

### Relationship Between CMR and miRNA Findings in Female MD-Carriers

When female MD-carriers with any pathological CMR findings (*N* = 14; 48%) were compared to those without (Table 4) (*N* = 15; 52%), the only significant difference in miRNA levels was the down-regulation of miR-29c that was undetectable in the plasma of female MD carriers with CMR abnormalities (*p* = 0.006). When selectively comparing female MD carriers with vs. without functional CMR abnormalities only or those with vs. without structural CMR abnormalities only (Tables 5, 6), again the down-regulation of miR-29c was the only significant difference in circulating miRNA expression levels. On the other hand, when including also the controls, there was no significant difference in miR-29c levels between carriers with cardiac involvement and healthy females (Figure 1B).

**Table 4:** Serum miRNA results in MD-carriers with normal vs. pathological CMR results.

miRNA	Normal CMR	Abnormal CMR	*p*-value
serum levels^∗^ (×10^3^)	*N* = 15	*N* = 14	
-206	33.76 (6.91–62.05)	52.69 (20.43–80.66)	0.38
-144	0.00 (0.00–0.00)	0.00 (0.00–0.00)	0.91
-146b-5p	3.87 (0.0–76.41)	0.00 (0.00–206.72)	0.85
-15b	0.00 (0.00–10.75)	0.00 (0.00–17.82)	0.72
-195	9.19 (0.32–13.87)	16.44 (0.88–43.05)	0.25
-20b	30.63 (17.47–63.50)	39.06 (9.29–64.87)	0.85
-21-5p	20.47 (0.00–113.60)	0.00 (0.00–62.82)	0.27
-221	0.00 (0.00–15.99)	0.00 (0.00–63.10)	0.81
-222	1792.39 (310.93–8598.78)	4569.66 (1893.05–6667.01)	0.29
-26a	191.59 (24.59–453.25)	193.35 (127.63–439.35)	0.78
-29a	0.00 (0.00–1.41)	0.00 (0.00–0.00)	0.07
-29c	1.00 (0.16–2.34)	0.00 (0.00–0.00)	**0.006**
-342	1687.45 (236.79–4373.70)	3049.04 (1420.74–4265.11)	0.35
-378a-3p	2.45 (0.00–71.22)	12.02 (0.00–74.48)	1.00
-378a-5p	27.01 (1.49–171.84)	76.42 (32.47–167.93)	0.16
-451	123.50 (14.24–280.66)	152.21 (26.07–236.13)	0.95
-93	40.69 (6.75–65.91)	0.00 (0.00–25.63)	0.11

*^∗^Each miRNA serum level was normalized to miRNA-16 serum levels and thereafter multiplied by 10^*3*^ in order to increase readability. Bold values indicate significant *p* < 0.05*.

**Table 5:** Serum miRNA results in MD patients with normal vs. impaired systolic function.

miRNA serum levels^∗^ (×10^3^)	Normal LV and RV function *N* = 22	Impaired LV and/or RV function *N* = 7	*p*-value
-206	31.23 (8.04–65.90)	68.56 (20.55–92.40)	0.28
-144	0.00 (0.00–0.00)	0.00 (0.00–0.00)	0.94
-146b-5p	0.00 (0.00–56.64)	0.00 (0.00–390.38)	0.24
-15b	0.00 (0.00–12.88)	0.00 (0.00–9.85)	0.78
-195	8.89 (0.18–17.18)	24.97 (6.58–38.61)	0.18
-20b	33.27 (16.54–60.50)	42.21 (10.04–84.14)	0.90
-21-5p	13.67 (0.00–123.96)	0.00 (0.00–32.73)	0.30
-221	0.00 (0.00–30.93)	0.00 (0.00–45.61)	0.82
-222	2901.55 (1164.30–6479.11)	4332.03 (2965.73–7495.03)	0.30
-26a	192.44 (51.27–417.33)	164.56 (62.34–691.04)	1.00
-29a	0.00 (0.00–0.37)	0.00 (0.00–0.00)	0.30
-29c	0.67 (0.00–2.25)	0.00 (0.00–0.00)	**0.032**
-342	1962.41 (993.44–3903.07)	4095.88 (2243.90–5670.60)	0.17
-378a-3p	1.42 (0.00–79.78)	20.45 (1.79–97.97)	0.60
-378a-5p	53.09 (14.01–167.93)	81.87 (36.95–213.23)	0.33
-451	130.17 (7.12–230.18)	236.81 (77.62–310.74)	0.38
-93	31.82 (0.00–71.07)	0.00 (0.00–0.00)	0.10

*^∗^Each miRNA serum level was normalized to miRNA-16 serum levels and thereafter multiplied by 10^*3*^ in order to increase readability. Bold values indicate significant *p* < 0.05*.

**Table 6:** Serum miRNA results in MD-carriers with vs. without presence of LGE.

miRNA	LGE-negative	LGE-positive	*p*-value
serum levels^∗^ (×10^3^)	*N* = 16	*N* = 13	
-206	36.83 (7.01–70.41)	38.73 (20.30–69.85)	0.78
-144	0.00 (0.00–0.00)	0.00 (0.00–0.00)	0.98
-146b-5p	7.49 (0.00–94.15)	0.00 (0.00–224.40)	0.88
-15b	0.00 (0.00–12.52)	0.00 (0.00–13.13)	0.85
-195	7.87 (0.45–12.72)	20.50 (1.22–47.48)	0.16
-20b	29.76 (13.84–61.54)	42.21 (11.55–66.22)	0.88
-21-5p	25.52 (0.00–95.44)	0.00 (0.00–54.90)	0.17
-221	0.00 (0.00–11.62)	0.00 (0.00–65.12)	0.65
-222	1759.21 (401.67–6935.68)	4807.30 (1958.67–6881.28)	0.16
-26a	178.08 (32.66–349.34)	205.07 (124.69–474.63)	0.71
-29a	0.00 (0.00–1.39)	0.00 (0.00–0.00)	0.09
-29c	0.99 (0.07–2.30)	0.00 (0.00–0.00)	**0.017**
-342	1661.47 (313.16–3882.08)	3076.78 (1449.90–4321.52)	0.25
-378a-3p	9.37 (0.00–93.72)	3.59 (0.00–32.33)	0.59
-378a-5p	24.00 (1.61–165.49)	81.87 (46.04–170.52)	0.09
-451	133.75 (21.35–262.75)	149.18 (0.00–236.81)	1.00
-93	35.08 (0.00–60.74)	0.00 (0.00–34.17)	0.20

*^∗^Each miRNA serum level was normalized to miRNA-16 serum levels and thereafter multiplied by 10^*3*^ in order to increase readability. Bold values indicate significant *p* < 0.05*.

Moreover, in female MD-carriers, borderline significant correlations were found between plasma levels of miR-29c and LGE extent (Spearman-Rho -0.358, *p* = 0.057) and LV-EF (Spearman-Rho +0.352, *p* = 0.061), respectively.

### Assessment of Possible Predictors for CMR Abnormalities

**FIGURE 2:**
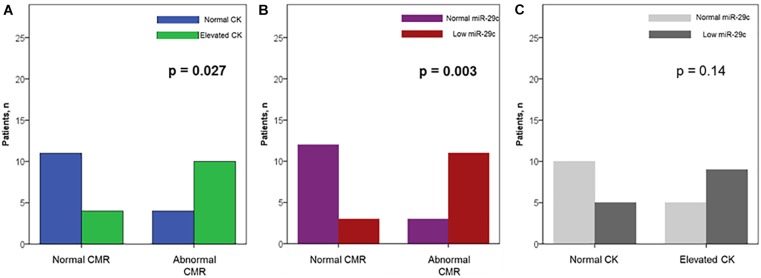
Prevalence of elevated plasma creatine kinase (CK) levels **(A)** and down-regulated circulating miR-29c **(B)** among carriers with and without abnormal CMR; **(C)** the relationship between an elevated CK and a down-regulated miR-29c in muscular dystrophy-carriers.

In order to identify potential predictors for the occurrence of CMR abnormalities (functional and/or structural), we first performed univariable logistic regression analyses for a series of parameters, including miR-29c (Table 7). In this analysis, a significant association with presence of an abnormal CMR was found for the following parameters: (a) CMR-derived LV-EDVi and LV-ESVi, (b) the presence of an elevated CK (Figure 2A) and (c) decreased levels of circulating miR-29c (Figure 2B). Subsequently, we introduced the four statistically significant variables into a multivariable regression analysis model. In this model, the only independent predictor for an abnormal CMR finding was Log_10_ miR-29c (OR 0.99, 95% CI 0.98–0.99, *p* = 0.037).

**Table 7:** Univariable analysis regarding predictors for an abnormal CMR in MD-carriers.

Variable (*N* = 29)	OR (95% CI)	*p*-value
Age	0.95 (0.89–1.01)	0.11
DMD carrier status (%)	4.19 (0.82–21.40)	0.09
LV-EDVi	1.06 (1.00–1.12)	**0.04**
LV-ESVi	1.13 (1.09–1.27)	**0.04**
LV-mass	1.00 (0.92–1.08)	0.90
Elevated hs-Trop^†^	1.08 (0.09–19.05)	0.96
Elevated CK^‡^	6.88 (1.35–35.06)	**0.020**
Log_10_ miR-29c (×10^3^)^∗^	0.99 (0.98–0.99)	**0.010**

*^†^ >14 pg/ml. ^‡^ >190 U/l. ^∗^Each miRNA serum level was normalized to miRNA-16 serum levels and thereafter multiplied by 10^*3*^ in order to increase readability. Bold values indicate significant *p* < 0.05*.

### Circulating miR-29c for the Identification of Female MD Carriers With CMR Abnormalities

**FIGURE 3:**
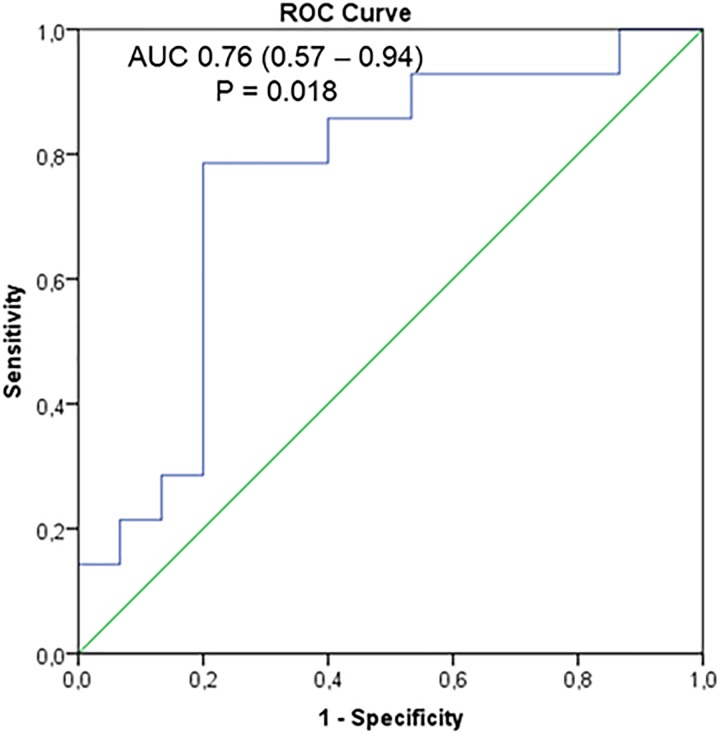
Individual receiver operating characteristics curves (ROC) for miR-29c plasma levels in the diagnosis of cardiac involvement among muscular dystrophy-carriers.

As shown in Figure 3, the individual ROC for the miR-29c, the only significantly (down-)regulated miRNA, in female MD-carriers with vs. without abnormal CMR findings revealed an area under the curve (AUC) of 0.76 (0.57–0.94, *p* = 0.018). The sensitivity, specificity and overall accuracy were 79, 80 and 80% for a plasma miR-29c cut-off value of 0.05 × 10^-3^. On the other hand, the AUC for an elevated serum CK value to identify a pathological CMR finding was 0.72 (0.573–0.92, *p* = 0.040), with 71, 73 and 72%, sensitivity, specificity and overall accuracy. All four female MD-carriers who demonstrated CMR abnormalities despite normal serum CK values showed plasma miR-29c levels “below” our cut-off, whereas all female MD carriers with abnormal CMR findings and a plasma miR-29c level “above” this cut-off (*n* = 3) had an elevated serum CK level (Figure 2C). Therefore, the presence of an elevated plasma CK and/or a downregulated miR-29c (<0.05 × 10^-3^) resulted in a further improved AUC value of 0.79 (0.62–0.97, *p* = 0.007) with 79, 80 and 80%, sensitivity, specificity and overall accuracy, respectively.

## Discussion

In the present study, the diagnostic value of circulating miRNAs for the detection and prediction of functional as well as structural cardiac abnormalities was assessed for the first time in female MD-carriers. Based on comprehensive CMR and epigenetic studies, we were able to show: (1) a series of circulating miRNAs are differently expressed in MD female carriers when compared to healthy volunteers; and (2) miR-29c is significantly down-regulated in female MD-carriers with cardiac involvement when compared to those without and independently predicts the presence of a pathologic CMR finding.

### Circulating miRNAs as Novel Biomarkers in Female MD-Carriers

We have recently shown that a series of circulating miRNAs are differently expressed in the plasma of DMD/BMD patients when compared to healthy male volunteers (Becker et al., 2016). Interestingly, those previously described miRNAs also comprise the currently detected six up-regulated (miR-206, -222, -26a, -342, -378a-3p and -378a-5p) as well as two down-regulated (miR-29a and miR-221) miRNAs, whose levels are significantly modified in female MD-carriers compared to healthy female controls. Noteworthy, changes in the above mentioned miRNAs have been previously associated either with heart disease and/or with skeletal myopathy in DMD/BMD (Becker et al., 2016). For example, serum miR-206 has already been proposed for the screening of MD carrier status and might even serve as a future therapeutic target in MD (Anaya-Segura et al., 2016; Amirouche et al., 2017). This is important considering that 10% of women with abnormally elevated serum CK turn out to be DMD carriers (Hoffman et al., 1992).

Regarding dissimilarities in miRNA expression, the levels of circulating miR-144 were significantly suppressed in female MD carriers when compared to both female controls and to MD males (*p* < 0.0001; significance maintained also on multiple comparison with *post-hoc* correction), with no gender-related differences in healthy individuals. Preclinical data suggest that miR-144 is involved in the regulation of reactive oxygen species formation and oxidative stress, and that decreases in miR-144 may exert protective cardiac muscle effects (31). However, in the present study no significant differences were observed in miR-144 expression between female MD-carriers with and without cardiomyopathy.

Surprisingly, the triad of significantly up-regulated miRNAs in MD patients with myocardial fibrosis (miR222; -26a; and -378a-5p) showed a similar upregulation when female MD-carriers were compared to female controls—but there was no significant variation in female MD-carriers with myocardial abnormalities (Becker et al., 2016). The exact reason for this unexpected observation is somewhat unclear and this result may even be obtained by chance due to the small sample size. However, the present data suggest that differences in the aforementioned miRNA triad signature are primarily due to differences in extra-cardiac tissues (most probably skeletal muscle) in female MD-carriers and theoretically could also be involved in one of the pathways responsible for the markedly milder MD phenotype in women compared to men (Florian et al., 2016).

### Circulating miRNAs in Female MD Carriers With and Without Cardiac Disease

When looking strictly at miRNAs associated with cardiac disease, the only significant difference in the circulating miRNAs tested was detected for miR-29c whose levels were undetectable in our (predominantly DMD) female carriers showing CMR abnormalities as reflected by myocardial fibrosis on LGE-CMR and/or mild impairments in ventricular systolic function. In principal, the miR-29 family is known to be involved in cell differentiation, proliferation, and apoptosis as well as to target components of the extracellular matrix and diseases including Huntington Disease and Rhabdomyosarcoma (van Re et al., 2008; Wang et al., 2008; Chuang et al., 2015). Moreover, it has been demonstrated that the miR-29 family comprises crucial players in the regulation of collagen and elastin mRNAs in the DMD fibrotic process in skeletal muscles and dystrophic muscle pathogenesis (Cacchiarelli et al., 2010; Wang et al., 2012). In contrast, miR29c belongs to microRNAs that are able to regulate the glucose transporter member 4 (GLUT4) of muscles (Esteves et al., 2017, 2018). This is interesting as GLUT4 is abnormally aggregating in the cytoplasm of DMD/BMD patients and linked to metabolic alterations such as obesity, hyperinsulinemia, and insulin resistance (Rodriguez-Cruz et al., 2015). In this context, miR-29c was also significantly downregulated in MD males vs. controls, but not in MD males with vs. without CMR signs of cardiomyopathy-, as shown recently (Becker et al., 2016). Interestingly, in our prior male MD study there was a BMD predominance and only 19% of the study population were DMD males. However, in the current MD-carrier study, 62% of our female patients were DMD-carriers. This difference in DMD percentage is important, since the miR-29 family is well-known to show a dystrophin-dependent upregulation as recently illustrated in human DMD myoblasts rescued for dystrophin synthesis by exon skipping gene therapy—a technique aiming to convert severe Duchenne into milder Becker forms (Cacchiarelli et al., 2010; Cazzella et al., 2012). Supporting this notion, the age- and DMD-matched males that were used for comparison in the present study, showed an even lower level of circulating miR-29c than the female MD-carriers (Supplementary Table 3). Unfortunately, the low number of MD males with normal CMR examinations (*n* = 5) prevented a proper and meaningful subgroup analysis regarding the association of cardiomyopathy and miR-29c. In summary, the downregulation of this microRNA in DMD/BMD carriers and patients seems to have multiple effects including muscle function, metabolic metabolism and dystrophic pathogenesis.

Most importantly, we showed that miR-29c downregulation was the only independent predictor—among several clinical, CMR and laboratory parameters, for identifying cardiac involvement in female MD carriers. Usually, cardiac involvement in female carriers is less frequent, milder and with a more “benign” course compared to their male MD counterparts (Hoogerwaard et al., 1999; Schade van Westrum et al., 2011; Mavrogeni et al., 2013; Florian et al., 2016). Nevertheless, an early diagnosis is critical since disease courses progressing to dilated cardiomyopathy are described (Melacini et al., 1998; Hoogerwaard et al., 1999; Finsterer and Stollberger, 2005; Schade van Westrum et al., 2011; Mavrogeni et al., 2013). The prevalence of cardiac and skeletal muscle symptoms among women with abnormal CMR was more than twice the number compared to those with normal CMR, yet without statistical significance. Unfortunately, these symptoms are usually mild and unreliable for deciding in which patient further work-up—including CMR imaging, will be necessary.

### Potential Clinical Implications

Considering the use of echocardiography screening, a large study by Mccaffrey et al. (2016) found a reduced LV-EF in only 13% of female MD carriers, with no correlation between echocardiographic findings and disease genotype, presence of muscle symptoms or CK level. Even when using the more precise CMR based quantification method, an impaired LV-EF is found in less than half of the female carriers with evidence of myocardial fibrosis, making this “functional” parameter an insensitive tool for an early diagnosis (Mavrogeni et al., 2013; Florian et al., 2016). Yet, we were able to show that an abnormally elevated total CK was associated with the presence of cardiomyopathy and identified it with a noteworthy accuracy, confirming the recently described link between skeletal and heart muscle damage in MD carriers (Giglio et al., 2014; Florian et al., 2016). Therefore, one may speculate that an elevated CK represents an important marker for selecting carriers at increased risk for cardiomyopathy, in whom further CMR imaging will be meaningful. On the other hand, the reported prevalence of CK elevation in carriers across studies is rather heterogeneous (from 32 to 100%), limiting its practical diagnostic usefulness (Mavrogeni et al., 2013; Giglio et al., 2014; Florian et al., 2016; Mccaffrey et al., 2016).

Taking into account that in relation to serum CK, (1) circulating miR-29c possessed a superior diagnostic accuracy and (2) adding information about miR-29c levels resulted in a slightly higher AUC, we propose a new algorithm for the early suspicion and subsequent diagnosis of cardiomyopathy in female MD carriers. Thus, regardless of age and disease subtype, all females with a confirmed DMD/BMD carrier status should undergo in addition to an initial basic cardiological evaluation (including transthoracic echocardiography), measurements of plasma CK and miR-29c levels. Thereafter, comprehensive CMR studies should be necessarily performed in those presenting with an elevated serum CK level AND/OR downregulated miR-29c (<0.05 × 10^-3^) (Kamdar and Garry, 2016). In the remaining MD carriers without CK elevation AND without a decrease in serum miR-29c, an additional CMR study is not mandatory based on our present preliminary data. Further investigations with longitudinal data have to verify such an approach and will help to elucidate whether the dynamics of the two biomarkers may also guide the timing and frequency of imaging follow-up.

### Limitations

The first limitation is that only a pre-defined number of miRNAs were tested, which have been selected a priori, based on already published data. A more comprehensive miRNA profiling approach may reveal even more complex changes in miRNA signature, particularly in carriers with cardiac involvement, with potential superior diagnostic and predictive value. Secondly, for the further implementation in clinical practice, our results need further verification and validation in larger cohorts, taking into account also clinical outcomes and the dynamic of biomarkers over time. Lastly, due to the small study size and the large number of encountered mutations, we limited our genotype-phenotype correlation to the presence of either a DMD or BMD carrier status (based on the available genetic diagnosis), without a proper genetic mutation analysis.

## Conclusion

In female MD carriers, down-regulation of plasma miR-29c relates to the presence of structural and/or functional CMR abnormalities and appears to be a promising novel biomarker, in addition to conventional CK plasma levels, for an early diagnosis of cardiomyopathy. Additionally, the similarities and dissimilarities in circulating miRNA signature between female MD carriers and their male counterparts promise to shed more light on the complex genotype-phenotype interactions in MD.

## Availability of Data and Materials

The datasets used and/or analyzed during the current study are available from the corresponding author on reasonable request.

## Author Contributions

AF participated in the CMR exams, carried out the data and statistical analysis, and wrote the initial draft version of the manuscript. AP participated in the CMR exams and in the analysis of the CMR data. RT was involved in the recruitment of study patients. SR participated in the CMR exams and in the analysis of the CMR data. MS and US critically reviewed the manuscript. ES and RT provided additional supervision and critically reviewed the manuscript. AY supervised the study, critically reviewed the manuscript, and drafted the manuscript. All authors read and approved the final manuscript.

## Conflict of Interest Statement

The authors declare that the research was conducted in the absence of any commercial or financial relationships that could be construed as a potential conflict of interest.

## Abbreviations

BMD: becker muscular dystrophy
CK: creatine kinase
CMR: cardiovascular magnetic resonance
DCM: dilative cardiomyopathy
DMD: Duchenne muscular dystrophy
hs-Trop: high-sensitive troponin
LGE: late-gadolinium-enhancement
LV: left ventricle
LV-EDV: left ventricular end-diastolic volume
LV-ESV: left ventricular end-systolic volume
LV-EF: left ventricular ejection fraction
MD: muscular dystrophy
NT-proBNP: brain natriuretic peptide
RV: right ventricle
RV-EDV: right ventricular end-diastolic volume
