# Supplementary material for: Identification of Cardiomyopathy-Associated Circulating miRNA Biomarkers in Muscular Dystrophy Female Carriers Using a Complementary Cardiac Imaging and Plasma Profiling Approach
Source: Front Physiol. 2018 Dec 21;9:1770. doi: 10.3389/fphys.2018.01770 (PMC6308188; doi:10.3389/fphys.2018.01770)
Supplement: Supplementary file 1 [file Table_1.pdf]

Supplementary table 1: Detailed list of dystrophin gene mutations

Sample	Sample ID for publication	Disease status	Dystrophin gene mutation
**RBK109-108**	**#2**	BMD	Deletion Exon 45-51
**RBK109-121**	**#3**	BMD	Deletion Exon 45-55
**RBK109-147**	**#4**	BMD	Deletion Exon 45-47
**RBK109-157**	**#5**	BMD	Deletion Exon 45-49
**RBK109-192**	**#6**	BMD	Splice mutation c.186+2T>C
**RBK109-20**	**#7**	BMD	Duplication Exon 29-34
**RBK109-270**	**#8**	BMD	c.5587-7T>A
**RBK109-279**	**#9**	BMD	Deletion Exon 17-18
**RBK109-37**	**#10**	BMD	Deletion Exon 45-48
**RBK109-82**	**#11**	BMD	Duplication Exon 2-7
**RBK109-87**	**#12**	BMD	Mutation Exon 8
**RBK109-111**	**#13**	DMD	Duplication Exon 18-29
**RBK109-112**	**#14**	DMD	Duplication Exon 18-29
**RBK109-118**	**#15**	DMD	Deletion Exon 48-50
**RBK109-127**	**#16**	DMD	Deletion Exon 48-54
**RBK109-140**	**#17**	DMD	Deletion Exon 45-50
**RBK109-141**	**#18**	DMD	Deletion Exon 9-28
**RBK109-167**	**#19**	DMD	Duplication Exon 42-50
**RBK109-177**	**#20**	DMD	Duplication Exon 42-50
**RBK109-246**	**#21**	DMD	Deletion Exon 46-55
**RBK109-247**	**#22**	DMD	Deletion Exon 46-55
**RBK109-45**	**#23**	DMD	Mutation Exon 55
**RBK109-56**	**#24**	DMD	Duplication Exon 8-11
**RBK109-60**	**#25**	DMD	Deletion Exon 8-28
**RBK109-62**	**#26**	DMD	Deletion Exon 8-28
**RBK109-63**	**#27**	DMD	Deletion Exon 8-28
**RBK109-74**	**#28**	DMD	Deletion Exon 43
**RBK109-94**	**#30**	DMD	Duplication Exon 5-18
**RBK109-95**	**#31**	DMD	Deletion Exon 51
