# Supplementary material for: Identification of Cardiomyopathy-Associated Circulating miRNA Biomarkers in Muscular Dystrophy Female Carriers Using a Complementary Cardiac Imaging and Plasma Profiling Approach
Source: Front Physiol. 2018 Dec 21;9:1770. doi: 10.3389/fphys.2018.01770 (PMC6308188; doi:10.3389/fphys.2018.01770)
Supplement: Supplementary file 2 [file Table_2.pdf]

Supplemental Table 2 (appendix). Serum miRNA results in male vs. female controls

miRNA serum levels* (*10^3)	Male controls N = 26	Female controls N = 24	p value
**Age, years**	36 ± 13	41 ± 11	0.15
**-206**	0.19 (0.12-0.64)	0.38 (0.13-0.87)	0.32
**-144**	13.16 (6.53-19.63)	12.65 (9.12-21.03)	0.92
**-146b-5p**	16.64 (6.86-33.02)	19.88 (6.41-42.27)	0.57
**-15b**	2.46 (0.81-3.93)	2.75 (1.24-8.49)	0.39
**-195**	13.63 (10.46-16.81)	13.46 (11.74-15.91)	0.88
**-20b**	35.95 (27.39-51.02)	41.51 (31.65-66.94)	0.11
**-21-5p**	9.58 (5.86-18.33)	10.55 (7.06-25.90)	0.60
**-221**	10.61 (7.73-17.12)	13.59 (0.89-35.06)	0.83
**-222**	79.22 (43.34-169.52)	95.65 (67.93-149.18)	0.61
**-26a**	49.40 (26.58-63.10)	52.50 (17.12-136.09)	0.71
**-29a**	0.07 (0.00-1.11)	0.93 (0.00-2.01)	0.45
**-29c**	0.56 (0.27-2.72)	0.83 (0.00-2.32)	0.38
**-342**	73.89 (45.30-171.19)	98.71 (66.63-142.78)	0.66
**-378a-3p**	0.00 (0.00-0.18)	0.00 (0.00-0.34)	0.56
**-378a-5p**	0.58 (0.30-1.79)	0.85 (0.48-1.55)	0.24
**-451**	144.45 (107.74-169.24)	124.83 (103.12-185.29)	0.61
**-93**	35.37 (25.60-49.70)	38.71 (27.66-46.52)	0.83

(*) – Each miRNA serum level was normalized to miRNA-16 serum levels and thereafter multiplied by 10^3 in order to increase readability.

Supplemental table 3 (Appendix). Serum miRNA results in male MD patients vs. MD-carriers

**miRNA serum levels* (*10³)**	**MD patients N = 25**	**MD-carriers N = 29**	**p value**
**Age, years mean +- sd**	36 ± 19	45 ± 14	0.09
**DMD, n (%)**	12 (48)	18 (62)	0.41
**-206**	15.54 (3.78-50.37)	38.73 (10.86-70.06)	0.15
**-144**	78.92 (0.00-228.67)	0.00 (0.00-0.00)	**0.001**
**-146b-5p**	7.41 (0.00-90.29)	0.00 (0.00-95.45)	0.78
**-15b**	0.37 (0.00-28.20)	0.00 (0.00-13.13)	0.27
**-195**	11.28 (0.57-28.38)	9.99 (0.58-20.91)	0.72
**-20b**	65.52 (19.11-83.77)	35.91 (11.55-66.22)	0.39
**-21-5p**	40.26 (0.00-108.25)	2.92 (0.00-77.28)	0.45
**-221**	0.00 (0.00-29.46)	0.00 (0.00-32.99)	0.60
**-222**	1616.92 (638.43-4732.47)	3972.80 (1489.47-6881.28)	0.11
**-26a**	257.99 (121.01-460.52)	191.59 (40.72-474.63)	0.48
**-29a**	0.00 (0.00-0.00)	0.00 (0.00-0.00)	0.54
**-29c**	0.00 (0.00-0.00)	0.09 (0.00-1.64)	**0.024**
**-342**	2069.11 (860.67-4114.63)	2633.27 (1194.51-4321.52)	0.53
**-378a-3p**	15.33 (0.00-69.46)	3.59 (0.00-88.53)	0.50
**-378a-5p**	21.14 (8.84-66.22)	53.57 (18.76-170.52)	0.18
**-451**	147.28 (30.24-243.25)	144.00 (0.00-244.83)	0.97
**-93**	26.19 (0.00-44.47)	13.50 (0.00-55.57)	0.85

(*) – Each miRNA serum level was normalized to miRNA-16 serum levels and thereafter multiplied by 10^3 in order to increase readability.
